# The Role of Bone-Derived Exosomes in Regulating Skeletal Metabolism and Extraosseous Diseases

**DOI:** 10.3389/fcell.2020.00089

**Published:** 2020-03-17

**Authors:** Huili Lyu, Ye Xiao, Qi Guo, Yan Huang, Xianghang Luo

**Affiliations:** Endocrinology Research Center, Department of Endocrinology, Xiangya Hospital of Central South University, Changsha, China

**Keywords:** exosome, skeletal metabolism, extraosseous diseases, bone tissue engineering, biomarker

## Abstract

Bone-derived exosomes are naturally existing nano-sized extracellular vesicles secreted by various cells, such as bone marrow stromal cells, osteoclasts, osteoblasts, and osteocytes, containing multifarious proteins, lipids, and nucleic acids. Accumulating evidence indicates that bone-derived exosomes are involved in the regulation of skeletal metabolism and extraosseous diseases through modulating intercellular communication and the transfer of materials. Following the development of research, we found that exosomes can be considered as a potential candidate as a drug delivery carrier thanks to its ability to transport molecules into targeted cells with high stability, safety, and efficiency. This review aims to discuss the emerging role of bone-derived exosomes in skeletal metabolism and extraosseous diseases as well as their potential role as candidate biomarkers or for developing new therapeutic strategies.

## The Introduction of Exosomes

**FIGURE 1:**
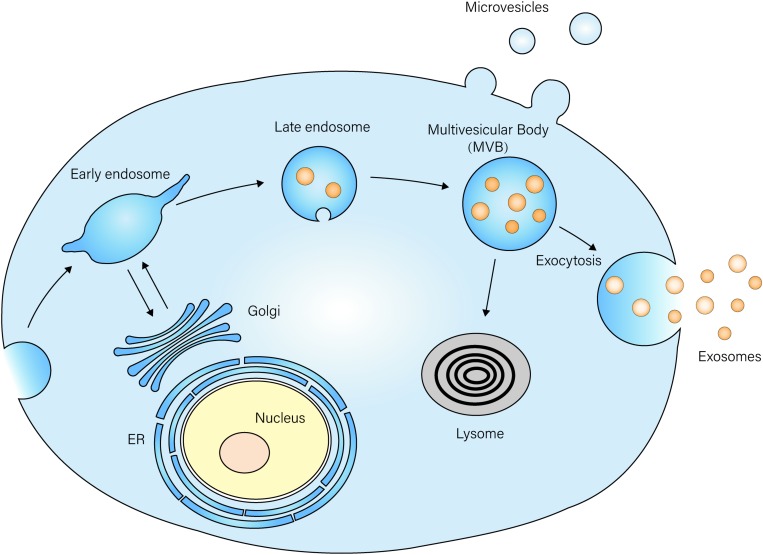
Exosome biogenesis and secretion The membrane of the late endosome buds inwardly to form luminal vesicles, which then transform into multi-vesicle bodies (MVBs). MVBs then fuse with the plasma membrane and release vesicles named exosomes into the extracellular space. MVBs can also be fused with lysosomes, and they degrade vesicles inside.

Exosomes, first discovered in the 1980s, refer to extracellular vesicles with a diameter of 30–100 nm, and they were thought to be involved in the selective release of the transferrin receptor during maturation of sheep reticulocytes (Pan and Johnstone, 1983; Johnstone et al., 1987). Despite existing in reticulocytes, exosomes were found to be released by a variety of other cells, including lymphocytes (Guay et al., 2019), dendritic cells, platelets, mast cells (Cheung et al., 2016), neurons, macrophages (Bourdonnay et al., 2015), mesenchymal stem cells (MSCs), intestinal epithelial cells (IECs), and so on (van Niel et al., 2001; Clayton et al., 2005; Taylor and Gerçel-Taylor, 2005; Raposo and Stoorvogel, 2013). Exosomes initiate formation when the inner membrane of the endosomes bud inwardly to form luminal vesicles, which then transform into multivesicular bodies (MVBs). These MVBs can either be fused with lysosomes to degrade themselves, or they can release vesicles, named exosomes, after fusion with the plasma membranes (Figure 1) (Simons and Raposo, 2009).

It was indicated that lipids are enriched on the surfaces of exosomes, including cholesterol, sphingomyelin, and ceramides. Furthermore, exosomes contain numerous biological molecules, such as proteins, enzymes, and microRNAs (miRNAs) (Valadi et al., 2007), which mediate intercellular communication and play an important role in the physiological and pathological processes (Kowal et al., 2014; Janas et al., 2015). Exosomes secrete complex content that depends not only on the cell type but also the microenvironment, such as mechanical properties, cellular PH, biochemical stimuli, and hypoxia (Choudhry and Harris, 2018). While previous research has shown that exosomes originating from different cells contain a specific subset of endosome-related proteins, they are involved in MVE biogenesis (e.g., Alix and Tsg101), membrane transport, and fusion (e.g., Rab GTPases, Annexins, and flotillin) (Colombo et al., 2014); among these actions, ubiquitous proteins like tetraspanins (CD9 and CD81), heat shock proteins (HSP70 and HSP90), and tumor-susceptibility gene 101 (Tsg101) are frequently used as markers of exosomes (Bjørge et al., 2017).

Exosomes have been widely investigated because of their multiple functions in diverse physiological process and diseases. As one of the most important factors in the paracrine regulation mechanism, exosomes can directly participate in signaling communication between cells. Dai et al. (2019) elucidated that prostate cancer (PCa)-derived exosomes could promote tumor growth and premetastatic niche formation in bone through transforming PKM2 into BMSCs. Alveolar macrophages (AMs) were found to secret exosomes containing the SOCS1 protein, which could be taken up by alveolar epithelial cells (AECs) and can inhibit STAT1 activation, leading to downregulation of inflammatory signaling both *in vitro* and *in vivo* (Bourdonnay et al., 2015). In addition, the microvesicles—released from primary lung epithelial cells induced by hyperoxia—containing hnRNPA2B1-associated miRNAs could be delivered into a macrophage and stimulate inflammation (Lee et al., 2019). Taken together, these experiments illustrated the essential role of exosomes in bilateral actions between AMs and AECs. Besides, the FasL-positive microvesicles released by melanoma cells were proven to induce the apoptosis of Jurkat and lymphoid cells, through which a tumor may escape from the effect of the immune system (Andreola et al., 2002). It can be seen that the exosomes derived from multiple cells are able to transfer different molecules, proteins, RNAs, and therefore have a significant effect on recipient cells.

## Techniques for Isolating Exosomes

To optimally understand and exploit the biological action and clinical application of exosomes, it is essential to isolate them from cell culture supernatants or primary body fluids. The exosomes originate from a wealth of sources, such as whole blood (Wu et al., 2017), menstrual blood (Dalirfardouei et al., 2018), urine (Street et al., 2017), cerebrospinal fluid (CSF) (Manek et al., 2018), milk (Leiferman et al., 2019), and so on. So far, a series of methods have been developed to isolate exosomes on the basis of size difference, molecular weight, density, certain surface markers, including differential ultracentrifugation (Raposo et al., 1996), density gradient ultracentrifugation (van der Pol et al., 2012), size based filtration, size-exclusion chromatography (Rood et al., 2010), immunoaffinity isolation (Kang et al., 2017), precipitation (Coumans et al., 2017; Li P. et al., 2017), field-flow fractionation (Zhang and Lyden, 2019), and so on. Generally speaking, every isolation technique exhibits its distinct advantages and disadvantages due to different experimental principles. Since exosomes have great potential and value in early clinical diagnosis, disease treatment, and prognosis evaluation, it is imperative to establish more user-friendly, efficient, and reliable technologies for the purpose of exosome isolation.

## The Characteristics and Contents of Bone-Derived Exosomes

In recent years it has been established that bone marrow stromal cells, osteoclasts, osteoblasts, and osteocytes can release exosomes that can not only regulate bone remodeling and skeletal disorders but can also participate in the progression of extraosseous diseases (Liu et al., 2017). Bone-derived exosomes contain a multitude of molecules, such as proteins and nucleic acids, that vary dynamically according to cell types as well as pathological and physiological conditions. In a recent study, researchers detected a total of 1,536 proteins contained in osteoblast-derived exosomes; they found that several valuable proteins involved in membrane trafficking and signaling pathways might be implicated in human bone diseases, including transforming growth factor beta receptor 3 (TGFBR3), lipoprotein receptor-related protein (LRP)6, bone morphogenetic protein receptor type-1 (BMPR1), and smad ubiquitylation regulatory factor-1 (SMURF1) (Ge et al., 2017). In addition, one proteomics profiling of exosomes from primary mouse osteoblasts revealed the difference in content between osteosomes under various differentiation statuses. To be more specific, 10 of the commonly expressed proteins were found to be increased more than five-fold in mineralizing (D24 osteosomes) primary mouse calvarial osteoblasts compared with proliferating osteoblasts (D0 osteosomes) (Bilen et al., 2017). Xu et al. (2014) tried to figure out the physiological role of exosomal miRNAs in osteoblast differentiation; they detected 79 miRNAs (∼8.84%) in exosomes isolated from BMSC culture supernatants and verified the presence of miRNA in exosomes during BMSCs osteogenic differentiation for the first time. Moreover, this study revealed differential expression of 14 exosomal miRNAs during osteogenic differentiation of human BMSCs; nine miRNAs (let-7a, miR-199b, miR-218, miR-148a, miR-135b, miR-203, miR-219, miR-299-5p, and miR-302b) were upregulated, and four miRNAs (miR-221, miR-155, miR-885-5p, miR-181a, and miR-320c) were downregulated (Xu et al., 2014). Another research study demonstrated that BMSC-derived exosomes could significantly reverse either S100- or LPS/ATP-induced injury in mice and hepatocytes. However, these protective effects were partly abolished with the involvement of the miR-223 inhibitor. This study firstly revealed the role of miRNA transferred by BMSCs-exo on liver injury caused by autoimmune hepatitis (Chen et al., 2018). Roccaro et al. (2013) found that BM-MSCs promoted the release and transfer of exosomes to MM (multiple myeloma) cells and detected a lower level of tumor-inhibiting factor miR-15a in MM BM-MSC versus normal BM-MSC-derived exosomes. Furthermore, a variety of oncogenic proteins and cytokines, which regulate adhesion and migration, were found to be enriched in MM BM-MSC-derived exosomes (Roccaro et al., 2013). In conclusion, the contents of bone-derived exosomes vary according to their different originators and microenvironments, which exert multiple effects on a serious of diseases, including skeletal disorders, prostate cancer, multiple myeloma, breast cancer, and so on.

## The Roles of Bone-Derived Exosomes in Skeletal Metabolism

The skeleton is considered to be an essential support organ that protects vital organs, stores minerals, and provides a mechanical bracket for body and movement (Han et al., 2018). Osteoblast-mediated bone formation and osteoclast-mediated bone resorption maintain a dynamic balance through signaling proteins, such as asephrin-Eph, which bone remodeling process occurs throughout the lifespan to ensure the integrity of the skeleton and its multiple functions (Zhao et al., 2006; Suchacki et al., 2017). It has also been proposed that osteocytes play essential roles in bone remodeling by affecting the activities of osteoblasts and osteoclasts (Tatsumi et al., 2007).

The molecular regulation mechanism of bone remolding is affected by many factors, such as age (Ambrogini et al., 2010), genes, proteins (Ishijima et al., 2001), hormone levels (Hayashi et al., 2019), enzymes (Jin et al., 2014), amino acids (Yu et al., 2019), and cytokines (Wang et al., 2015). For example, the bone-resorbing activity of osteoclasts was negatively regulated by a signal transducer and activator of transcription 5 (Stat5), which performs its functions through suppressing MAPK activity via regulation of Dusp1 and Dusp2 (Hirose et al., 2014). Researchers found that sphingosine-1-phosphate (S1P) is able to mediate the location of osteoclast (OC) precursors (OPs) between bone and blood through its receptors, and thereby influence the osteoclastogenesis and bone remolding (Ishii et al., 2010). Glucocorticoids are shown to induce bone loss through inhibiting osteoblast function and differentiation by targeting glucocorticoid receptors and suppressing AP-1-dependent cytokines (Rauch et al., 2010). Greenblatt et al. (2015) also found a key regulator of bone turnover—charged multivesicular body protein 5 (CHMP5); it can suppress the RANK-induced NF-κB signaling in osteoclasts and thereby dampen osteoclast differentiation.

An imbalance in bone metabolism regulation can lead to many diseases, including osteoporosis, which is characterized by age-related bone loss and increased fat formation (Moerman et al., 2004), high bone mass (HBM) resulting from abnormally increased bone formation (Yang et al., 2019), osteoarthritis, which is marked by progressive degeneration of articular cartilage (Rahmati et al., 2017), and so on. For example, the follicle-stimulating hormone (FSH) could promote activation of osteoclasts and enhance bone resorption through several signaling pathways, such as NF-kB, thus leading to a high risk of osteoporosis associated with menopause (Sponton and Kajimura, 2017). In recent years, scholars have made remarkable progress in the study of bone remodeling and related diseases as well as the role exosomes play in bone remolding, and this is attracting more and more attention.

### BMSC-Derived Exosomes

There is a class of stromal stem cells with the capacity for autologous renewal in bone marrow named BMSCs. They can differentiate into osteoblasts, chondrocytes, and adipocytes (Fan et al., 2017; Li et al., 2018), and they can thereby influence bone formation, maintenance, and reconstruction (Nakajima et al., 2018). Emerging evidence suggests that BMSCs have the ability to repair bone tissue damage and promote bone regeneration, but the specific mechanism involved in this has not been clarified. It has been reported that transplanted MSCs are not able to exist *in vivo* permanently, but the therapy effect still persists even after MSCs elimination. Recently, scientists have found that this bone-protective effect may be related to paracrine vesicles, much like exosomes secreted by BMSC (Ng et al., 2015).

One study revealed that exosomes isolated from bone-marrow-derived MSCs could rescue the retardation of fracture healing in CD9^–/–^ mice. The mechanism in this process is more than the recruitment of stem cells or progenitor cells by cytokines, but it also involves the induction of osteogenesis and angiogenesis partly regulated by exosomal miRNAs (Furuta et al., 2016). Fang et al. (2019) demonstrated that BMSC Exos significantly reverse the decreased osteogenic differentiation of BMSCs in steroid-induced femoral head necrosis (SFHN), and they then detected a total of 84 genes and 20 differentially expressed genes (DEGs), which consist of 11 upregulated and nine downregulated genes. Among these, DEGs, Bmps, Mmp9, and Sox9, which are related to regulating the immune system processes and the BMP/TGF-β pathway, may play important roles in the pathogenetic process of SFHN (Fang et al., 2019).

The exosomes’ potential osteogenic capacity in bone regeneration was declared by Narayanan et al. (2016). They performed a series of studies to investigate how osteogenic exosomes derived from HMSCs could be endocytosed by primary HMSCs and could upregulate the expression of BMP9 and TGF-β1, which are both potent molecules in osteogenic differentiation (Luther et al., 2011; Kuroda et al., 2012). In addition, the *in vivo* experiments also indicated that both the regular exosomes and osteogenic exosomes can promote the osteogenic differentiation of HMSCs and matrix mineralization, while the osteogenic exosomes have a higher potential to induce better vascularization and calcium phosphate nucleation (Narayanan et al., 2016). Qin et al. (2016) found that exosomes isolated from BMSCs could be internalized into osteoblasts and enhance the expression of osteogenic genes. To investigate the mechanism by which exosomes promote osteogenic differentiation, they detected the miRNA in exosomes by miRNA sequencing and found highly expressed miR-27a, miR-206a, and miR-196a, among which miR-196a has the greatest potential in functional testing (Qin et al., 2016). Liu et al. (2015) indicated that transplantation of BMMSCs could rescue osteopenia in Fas-deficient-MRL/lpr mice through secreting exosomes, which were found to transfer Fas to recipient MRL/lpr BMMSCs, reduce their miR29b levels, enhance osteogenic differentiation *in vitro*, and promote bone formation *in vivo*.

Despite the critical effect in bone remolding, several studies have confirmed that BMSC-derived exosomes have the ability to reduce cartilage destruction and promote cartilage repair (Pourakbari et al., 2019). Osteoarthritis (OA) is a kind of rheumatic disease characterized by degeneration of articular cartilage and osteophyte formation (Findlay and Kuliwaba, 2016), and previous research has suggested that bone marrow-derived MSCs could be applied to treating OA and cartilage lesions in animals and humans (Nejadnik et al., 2010; Xie et al., 2012). Recently, accumulating evidence has implied that exosomes isolated from BMSCs function as vital media to elicit protective responses in cartilage. Cosenza et al. (2017) identified that BM-MSCs-derived exosomes could protect cartilage and bone from degradation through reducing apoptosis of chondrocytes, inhibiting macrophage activation, and promoting polarization of the M2 macrophage. Toh et al. (2017) proposed that the mechanism through which MSC exosomes modulate cartilage repair might rely on their ability to restore homeostasis in bioenergetics, cell number, and immunoregulation. Researchers addressed how MSCs are able to secrete miRNA-enriched exosomes to facilitate intercellular communication (Chen et al., 2010), and it is thus reasonable to conclude that exosomal miRNAs may be essential in cartilage differentiation. For instance, exosomal miRNA-23b could induce chondrogenic differentiation of hMSCs via inhibiting the expression of target PKA (Ham et al., 2012). Besides, Ning et al. (2013) found that miR-92a deficiency could cause a reduced number of chondrogenic progenitors, impaired chondrogenic differentiation, and pharyngeal cartilage defects in zebrafish. The mechanisms involved in the effect of miR-92a on chondrogenic differentiation rely on its regulation of Bmp signaling through targeting nog3 mRNA (Ning et al., 2013).

### Osteoclast-Derived Exosomes

**FIGURE 2:**
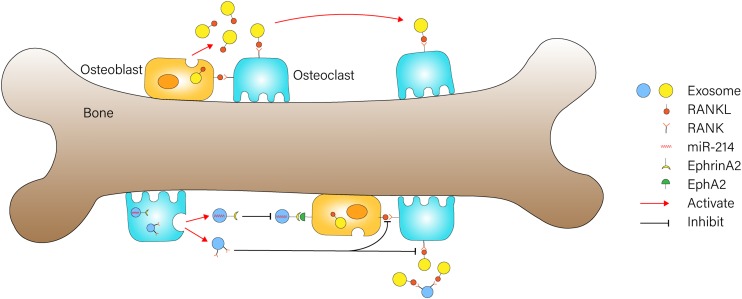
Exosome-mediated communication between osteoblasts and osteoclasts Osteoblast-derived exosomes containing RANKL could stimulate osteoclast differentiation through binding to RANK on the surface of osteoclasts. RNAK-enriched exosomes from osteoclasts competitively bind to RANKL and prevent activation of the RANK signaling pathway in osteoclasts. miR-214-containing exosomes from osteoclasts could be transferred into osteoblasts via ephrinA2/EphA2 recognition to inhibit osteoblast function.

Osteoclasts are giant multinucleated cells originating from mononuclear macrophage precursor cells, and they are mainly responsible for bone resorption *in vivo* (Nevius et al., 2015). Osteoclasts participate in the occurrence and development of many bone diseases, such as osteoporosis (Novack, 2007; Hsu et al., 2011), rheumatoid arthritis, and so on. Sun et al. (2016) showed that osteoclasts could secrete miR-214-containing exosomes, which are transferred into osteoblasts via ephrinA2/EphA2 recognition to inhibit osteoblast function (Figure 2). Besides, the level of miR-214 is measured to be higher in the serum exosomes of osteoporotic patients and mice compared with healthy ones, which might be a potential biomarker for osteoporosis (Sun et al., 2016). Moreover, the work of Zhao et al. (2015) demonstrated that miR-214 plays a catalytic role during RANKL-induced osteoclast differentiation through the PI3K/Akt signaling pathway by targeting phosphatase and tensin homolog (Pten). They also established Acp5-miR-214 transgenic (OC-TG214) mice to explore the effect of miR214 *in vivo*, and the results indicated that the upregulation of miR-214 in osteoclasts induced lower levels of Pten protein, higher activity of osteoclast bone resorbing, and poorer bone mineral density (BMD) (Zhao et al., 2015). Researchers tested the regulation of osteoclast formation in 1,25(OH)_2_D_3_-stimulated mouse marrow; they firstly found that osteoclast precursor-derived exosomes significantly stimulated formation of osteoclasts, while exosomes from mature osteoclasts suppressed osteoclastogenesis. Next, the presence of enriched RANK was found in exosomes from osteoclasts. The RANK depletion of osteoclast-derived exosomes reduced their effect of inhibiting osteoclastogenesis. These results revealed that RNAK contained in exosomes might be the active agent that competitively binds to RANKL and prevents stimulation of the RANK signaling pathway in osteoclasts, and it thus plays a greater inhibitory role in osteoclastogenesis (Figure 2) (Huynh et al., 2016). Li et al. (2016) delineated the role of exosomal miR-214-3p in the intercellular communication between osteoclasts and osteoblasts, which could be transferred from osteoclasts to osteoblasts to inhibit osteoblastic bone formation. More importantly, osteoclast-targeted inhibition miR-214-3p effectively reversed the suppression of osteoblast activity and facilitated bone formation, and this is suggestive of which might be a potential therapeutic method for bone loss (Li et al., 2016).

### Osteoblast-Derived Exosomes

Osteoblasts are derived from bone marrow MSCs with multidirectional differentiation potential, and they play a critical role in the synthesis, secretion, and mineralization of the bone matrix through its capacity to secrete glycoprotein and collagen. BMSCs first differentiate into osteoprogenitor cells, then turn into osteoblast precursors, and finally transform into osteoblasts (Garg et al., 2017), and this process is regulated by miRNAs (Li et al., 2009), proteins (Zou et al., 2013), signaling pathways, and so on. One *in vitro* research conducted by Cui et al. (2016) demonstrated the intercellular positive feedback mechanism between mineralizing osteoblasts (MOBs) and bone marrow stromal cells (ST2 cells). Their experiment results indicated that MOB-derived exosomes could be incorporated into ST2 cells and significantly promote their osteogenic differentiation; during this process, 91 miRNAs were detected as being overexpressed and 182 miRNAs were downregulated. Additionally, the analysis of the targeting genes and pathway networks of these miRNAs revealed that the pro-osteogenic function of MOBs-derived exosomes may partially depend on activation of the Wnt signaling pathway through downregulating Axin1 expression and upregulating β-catenin expression (Cui et al., 2016). Ge et al. (2017) identified 1,536 proteins in osteoblast-derived exosomes and sorted out several pivotal proteins that are closely related to bone diseases through network and pathway analyses. Deng et al. (2015) indicated that osteoblast-derived microvesicles can transfer RANKL proteins into osteoclast precursors and promote their differentiation into osteoclasts through activating RANKL–RANK signaling, which revealed a new mechanism involved in communication between osteoblasts and osteoclasts (Figure 2). Then, they conducted a series of experiments to further explore the role of microvesicles in bone metabolism by using imipramine, which was found to block microvesicles generation (Bianco et al., 2009). The *in vitro* results showed that inhibition of microvesicles generation from osteoblasts leads to significant suppression of osteoclast differentiation. Besides, the *in vivo* experiments revealed that OVX mice treated with imipramine exhibit superior properties in bone mineral density, bone volume, trabecular number, and thickness, suggesting that imipramine could prevent progression of bone loss caused by estrogen deficiency due to its function of blocking microvesicles generation (Deng et al., 2017). Through literature review and analysis, Xie et al. (2017) summarized the role of a number of mineralizing MC3T3 cell-derived exosomes in bone remolding. To be specific, miR-30d-5p, miR-133b-3p, and miR-140-3p were found to inhibit osteoblast differentiation, while let-7, miR-335-3p, miR-378b can promote osteoblast differentiation (Xie et al., 2017).

### Osteocyte-Derived Exosomes

Bone tissue is formed by the bone matrix and osteocytes originating from osteoblasts, which are widely distributed in the bone matrix and are tightly connected through the bone lacuna–tubule network system (Bonewald, 2007). Sato et al. firstly indicated the relationship between osteocyte exosomes and circulating exosomes in the serum. It was hinted that 12 miRNAs levels were significantly decreased in the circulating exosomes of OL (osteocyte less) mice plasma in contrast with control mice plasma. Therefore, it could be concluded that osteocytes release exosomes containing specific miRNAs that are transferred into circulating (Sato et al., 2017). The osteocyte-derived exosomes have been proven to mediate muscle–bone communication, and Qin et al. (2017) established that osteocyte-derived exosomes, which express the downregulation of miR-218 after myostatin treatment, can be incorporated into ostoblastic cells and suppress osteoblastic differentiation through downregulation of Wnt signaling; this can be reversed by expression of exogenous miR-218, suggesting the therapeutic potential of miR-218 in osteocytes for the treatment of bone disorders. In addition, the work of Zhang et al. (2014) confirmed the relationship between miR-218 and Wnt/β-catenin signaling in the osteogenic differentiation of human adipose tissue-derived stem cells (hASCs). Their *in vitro* experiments elucidated that overexpression of miR-218 activates the Wnt signaling pathway via directly downregulating SFRP2/DKK2 levels and then promotes hASCs osteogenic differentiation, whereas upregulation of Wnt/β-catenin signal can enhance the expression of miR-218 (Zhang et al., 2014). It has been proven that Wnt signaling acts as a positive regulator in osteoblast differentiation and regulation of bone mass (Esen et al., 2013). Taken together, miR-218 functions as a signal amplifier in the complex feed-forward regulatory circuit to activate hASCs osteogenic differentiation (Zhang et al., 2014), which further demonstrated the potential application prospect of exosomal miR-218 in bone regeneration.

## The Effect of Bone-Derived Exosomes in Extraosseous Diseases

Recent studies have shown that bone-derived exosomes not only participate in regulating bone remodeling in the bone microenvironment but also act as vital communication media in multiple biological processes, including antigen presentation, apoptosis, inflammation, cancer progression, and so on (Gurunathan et al., 2019). As a result, the multiple roles of bone-derived exosomes in distant tissues and extraosseous systems attracted much attention.

### Role of Bone-Derived Exosomes in Cancer

So far, people have tried many ways to explore the mechanism of cancer and conquer oncotherapy. There is no denying that we have a deeper understanding of cancer in multiple areas, such as immune responses (Fritz and Lenardo, 2019), inflammation (Ritter and Greten, 2019), lipid metabolism (Snaebjornsson et al., 2020), and so on, but there is still no proper method that could cure cancer without side effects. It is therefore interesting that bone-derived exosomes were found to participate in the progression of cancer in terms of regulating gene expression, angiogenesis, migration, and proliferation. The work of Naseri et al. (2018) indicated that BMSCs-derived exosomes (MSCs-Exo), which are loaded with LNA-anti-miR-142-3p, can deliver their cargos into 4T1 and TUBO breast cancer cells—leading to downregulation of miR-142-3p and overexpression of tumor suppressor genes—and thereby exert an efficient anti-tumor function. Additionally, their *in vivo* experiments revealed that the mice treated with LNA-miR-142-3p inhibitor-loaded MSCs-Exos showed reduced tumor volume and growth rate. These results confirmed the efficiency of transferring anti-miR molecules like LNA-anti-miR-142-3p into target tissues via MSCs-Exos and also provided new outlooks in terms of oncotherapy (Naseri et al., 2018). In another study, researchers explored how exosomes isolated from human bone marrow mesenchymal stem cells (hBMSCs) can promote migration and proliferation of osteosarcoma and gastric cancer cells through activating the Hedgehog signaling pathway (Qi et al., 2017). Additionally, it is reported that the Hedgehog signaling pathway, an essential regulator in maintaining tissue polarity and cell differentiation, plays a significant role in multiple cancers, including most basal cell carcinomas (BCCs) and extracutaneous tumors (Yang et al., 2010). Bliss et al. (2016) tried to investigate the mechanisms by which MSCs communicate with BCCs (breast cancer cells) and promote dormancy of human breast cancer cells in bone marrow. They demonstrated that MSCs primed by cancer cells can release exosomes containing miR-222/223, which in turn favor their survival and enhance drug resistance. Moreover, the treatment of MSCs transfected with anti-miR222/223 facilitated chemosensitivity of dormant BCCs in mice (Bliss et al., 2016).

Zhu et al. (2012) figured out another mechanism by which BMSCs-derived exosomes promote tumor growth *in vivo*. They detected higher expression of VEGF and CXCR4 in tumor cells treated with BMSC-exosomes, suggesting that BMSC-exosomes could enhance angiogenesis and provide tumors with a richer blood supply and therefore promote their proliferation and growth. However, the results of another experiment are different from these. Bruno et al. (2013) found that human hepatocellular carcinoma cells (HepG2), human ovarian cancer cells (Skov-3), and Kaposi’s sarcoma cells incubated with BMSC-derived microvesicles exhibited lower proliferation ability, higher expression of negative regulators of the cell cycle, and an increased number of cells staying in the G0/G1 phase. They also observed the effect of microvesicles on growth of implanted tumors in mice, and the data indicated that BMSC-derived microvesicles could inhibit tumor growth and increase necrosis areas in tumors (Bruno et al., 2013). These opposing conclusions could be derived due to the occasion of MSCs or microvesicles treatment (Klopp et al., 2011). The injection of BMSC-derived extracellular vesicles (EVs) into established tumors could inhibit tumor growth, while the growth of tumors in the early stages treated with EVs could be facilitated.

### Role of Bone-Derived Exosomes in Neurologic Diseases

Researchers demonstrated that exosomes isolated from MSCs can mediate the transfer of miR-133b into astrocytes and neurons, inducing increased axonal plasticity and neurite remodeling in the ischemic boundary zone (IBZ), and thereby lead to functional recovery in rats subjected to middle cerebral artery occlusion (MCAo) (Xin et al., 2012, 2013). Besides, miR-133b + MSC treatment dramatically downregulated the expression of the connective tissue growth factor (CTGF) and the Ras homolog gene family member A (RhoA) (Xin et al., 2013), which are relevant to central nervous system (CNS) repair, post-injury restructuring (Hertel et al., 2000) and regulation of axonogenesis (Hall and Lalli, 2010). In another study, Nakano et al. (2016) found that rat BM-MSC-derived exosomes can ameliorate learning and memory impairment in STZ-diabetic mice through suppressing oxidative stress and recovering decreased synapse numbers, and this process might rely on the internalization of exosomes into astrocytes and neurons, causing recovery of damaged astrocytes and neurons. To further investigate the specific miRNAs or proteins that participated in improving diabetes-induced cognitive impairment, they conducted a series of experiments. The results proposed that the oversecretion of exosomal miR-146a due to endogenous bone marrow-derived MSCs within the condition of an enriched environment (EE) plays a key role in restraining astrocytic inflammation and preventing cognitive impairment in diabetic rats (Kubota et al., 2018). Mead and Tomarev (2017) demonstrated that BMSC-derived exosomes can be efficiently integrated into target retinal ganglion cells (RGC), thus promoting the recovery of the RGC function followed by optic nerve crush (ONC) through enhanced regeneration of RGC axons and inhibiting RGC loss in an miRNA-dependent manner. It is worth mentioning that the neuroprotective effect of BMSC-derived exosomes on RGC after ONC is significantly more potent than simple BMSC treatment (Mead et al., 2013; Mesentier-Louro et al., 2014), and this is probably due to their capacity to integrate into the retina and retain high doses. In conclusion, BMSC-derived exosomes exert a significant effect on promoting nerve repair and functional recovery, indicating their potential in the field of regeneration and remodeling of nervous system (Qing et al., 2018).

### Role of Bone-Derived Exosomes in Nephropathy

It has been reported in previous researches that BM-MSCs play a key role in protecting kidney from injury (Morigi and De Coppi, 2014), although the mechanisms are not entirely elucidated. Recently, several reports have demonstrated the critical roles of BM-MSC-derived exosomes in renal injury. Wang et al. (2019b) elucidated that BMSC exosomes protect renal tubular epithelial cells (NRK-52E) against apoptosis caused by ischemia reperfusion at the early reperfusion stage through transfer of exosomal miR-199a-5p from BMSCs into NRK-52E, leading to the suppression of endoplasmic reticulum (ER) stress in target cells by targeting the binding immunoglobulin protein (BIP) (Wang et al., 2019b). In another experiment, exosomes derived from MSC-CM were found to play a significant role in preventing diabetic nephropathy by inhibiting apoptosis of tubular epithelial cells (TECs) and reversing decreased tight junction protein ZO-1, thereby enhancing the barrier function of renal tubules (Nagaishi et al., 2016). In the model of kidney injury induced by ischemia reperfusion, BMSC-derived exosomes could inhibit the apoptosis of tubular cells and enhance their proliferation, thus protecting the kidney against AKI and CKD (Gatti et al., 2011).

Bruno et al. (2009) reported that the incorporation of exosomes isolated from bone marrow MSCs into tubular epithelial cells (TECs) can exert proliferative and antiapoptotic effects *in vitro* and significantly alleviate lesions of glycerol-induced AKI in SCID mice. Moreover, they provided evidence that the effects mentioned above were completely abolished by RNase treatment, indicating that the horizontal transfer of mRNAs contained in exosomes appears to be a requirement of their protective functions (Bruno et al., 2009). Similarly, one study revealed that BM-MSC-derived exosomes have a positive effect on renal PTECs (proximal tubular epithelial cells) after ATP depletion injury, which could inhibit PTECs apoptosis and enhance transespithelial resistance (TER), at least partly, via miRNAs transfer or through transcription modulation stimulated by exosomes. The GO analysis of modulated miRNAs confirmed that several upregulated genes in the condition of ATP depletion injury were suppressed after exosome treatment, including SHC1 (Src homology 2 domain containing transforming protein 1), caspase-3, caspase-7, and SMAD4 (SMAD family member 4) genes (Lindoso et al., 2014).

### Role of Bone-Derived Exosomes in Cardiac Diseases

Previous studies have proposed that BMSCs can protect cardiomyocytes from apoptosis and prevent LV (left ventricular) remodeling after MI through paracrine signaling (Uemura et al., 2006), suggesting the potential role of BMSCs in cardiac diseases. Researchers have carried out numerous experiments to investigate the underlying mechanism involved in their cardioprotective effects. Recent studies turned to focus on exosomes which are known as key transporters of paracrine factors (Gartz and Strande, 2018) and revealed the emerging role of exosomes as candidate for treatment of cardiac diseases. One research completed by Ma et al. (2018) confirmed that BMSC-derived exosomes loaded with miR-132 could be taken up by HUVECs (Human umbilical venous endothelial cells), causing overexpression of miR-132 in HUVECs, and they could thereby enhance angiogenesis *in vitro* and promote recovery of cardiac function in an AMI (acute myocardial infarction) model. To clarify the mechanism by which miR-132 exosomes modulate angiogenesis, they further measured miR-132 target gene RASA1 (Lei et al., 2015). Their data indicated that downregulated RASA1, in response to increased miR-132, can stimulate neovascularization and maintain the cardiac function *in vivo* (Ma et al., 2018). Feng et al. (2014) demonstrated that BMSC-derived exosomes could mediate the transfer of highly expressed miR-22 in the condition of ischemic preconditioning (IPC) from BMSCs into cardiomyocytes, leading to reduced apoptosis and cardiac fibrosis after myocardial infarction by downregulating the expression level of target Mecp2. Shao et al. (2017) compared the efficiency of bone marrow-derived MSCs and MSC-Exo in myocardial infarction recovery for the first time. The results displayed that MSC-Exo gained an advantage over MSCs in preserving myocardial function after infarction through inhibiting inflammation, suppressing fibrosis, promoting proliferation, and reducing apoptosis. Moreover, the sequencing analysis of miRNA indicated that MSC-Exo and MSCs possessed a similar miRNA profile, including downregulated miR-130, miR-378, and miR-34 as well as upregulated miR-29 and miR-24, suggesting that MSC-Exo could be considered as the replacement for MSCs to develop innovative therapies for myocardial infarction.

### Role of Bone-Derived Exosomes in Cutaneous Repair

During the cutaneous repair process, a variety of cells, growth factors, and the extracellular matrix cooperate to replace dead cells and reconstruct damaged tissues, in which the proliferation of fibroblasts, angiogenesis, skin cell proliferation, and re-epithelization are extremely critical (Wu et al., 2018). In a study conducted by McBride et al. (2017), Wnt3a was detected as being tethered with human BM-MSC exosomes exteriorly. Their *in vitro* results further implied that BM-MSC exosomes significantly promoted dermal fibroblast proliferation, migration, and angiogenesis, which simulative function depends heavily on CD63+ exosomes and Wnt co-receptor lipoprotein-related proteins (LRP6) (McBride et al., 2017). Shabbir et al. (2015) found that BM-MSC exosomes can be incorporated into fibroblasts and stimulate their growth and migration in a normal and diabetic chronic wound; they could also be uptaken by HUVEC cells and enhanced endothelial angiogenesis *in vitro*. Furthermore, BM-MSC exosomes can activate several pathways that play important roles in skin wound healing, such as AKT, ERK 1/2, and STAT3, and enhance the expression of various growth factors (Shabbir et al., 2015). Considering the important role of STAT3 signaling in wound healing (Dauer et al., 2005), they further investigated the content of exosomes and finally found STAT3 DNA binding activity in BMSC-derived exosomes.

### Role of Bone-Derived Exosomes in Metabolic Diseases

Nowadays, patients suffering from metabolic diseases are increasingly widespread through the world, including diabetes, obesity, hyperlipidemia, and so on (Ling and Rönn, 2019; Warshauer et al., 2020). Dysfunction of metabolism is found to greatly promote the occurrence and progression of these diseases (Newgard, 2017). For example, it is already known that the function of the pancreatic islet α cell is extremely important to glucostasis, and researchers have demonstrated that the a kind of pancreatic lncRNA called Paupar could activate Pax6 α-cell target genes and help to maintain glucose homeostasis; the deletion of Paupar in mice resulted in damaged α-cell function (Singer et al., 2019). Recently, researchers have demonstrated that exosomes isolated from BM-MSCs in aged mice could be incorporated into adipocytes, myocytes, and hepatocytes and reduce their insulin sensitivity through targeting SIRT1 (sirtuin 1). They also conducted miRNA microarray analysis and detected extremely high expression of miR-29b- 3p in exosomes from BM-MSCs of old mice. The injection of nanocomplexes mediating BM-MSCs-specific inhibition of miR-29b-3p could alleviate the aging-associated insulin resistance in mice, suggesting the potential therapeutic role of exosomal miR-29b-3p in aging-associated insulin resistance (Su et al., 2019).

## Bone-Derived Exosomes as a Source of Biomarkers for Disease Diagnosis

The content in exosomes are alterative according to the physiological and pathological state of cells; exosomes can thus reflect the physiological condition and disease development to some extent. For example, researchers analyzed human plasma EV proteomes in rest and exercise, and they found 322 differently expressed proteins, suggesting transient release of EVs into circulation in the condition of exercise (Whitham et al., 2018). Besides, exosomes can be easily collected because of their existence in blood, urine, milk, and so on (Mori et al., 2019). In previous studies, researchers have discovered the clinical value of miRNAs as potential biomarkers in some diseases. For example, the contents of two miRNAs that are elevated during osteoclastogenesis, miR16 and miR378, are found to be correspondingly higher in mice with a heavy bone metastatic tumor burden or in breast cancer patients, suggesting their potential as indicators for bone metastasis progression (Eii et al., 2013). At present, the proteins and nucleic acids contained in exosomes have been widely researched for their potential as biomarkers in clinical disease diagnosis (Wang et al., 2018).

Sato et al. (2017) firstly revealed the positive correlation between osteocytes and 12 miRNAs contained in circulating plasma exosomes, suggesting that osteocytes released exosomes that then transferred into blood. Muntión et al. (2016) isolated BM-MSC-derived exosomes from MDS patients and healthy donors (HD) and then performed microRNA expression arrays. The results demonstrated that 21 microRNAs in exosomes from MDS patients were overexpressed compared with HDs, among which the microR-10a and miR-15a were extremely high (Muntión et al., 2016). In MM patients, BMSCs release exosomes and deliver them into MM cells to regulate tumor progression. Compared with normal humans, exosomes isolated from patients’ BMSCs contain lower levels of tumor suppressor gene miR-15a and higher levels of oncogenic proteins, cytokines, and adhesion molecules (Roccaro et al., 2013). It has also been reported that elevated serum exosomal miR-214-3p is closely associated with reduced bone formation in elderly women with fractures and OVX mice, suggesting that there is great potential for the use of exosomal miR-214-3p in the diagnosis of osteoporosis (Li et al., 2016). Barrera-Ramirez et al. (2017) compared miRNA profiling of hMSC-derived exosomes from patients with acute myeloid leukemia (AML) patients and healthy people. They identified five differentially expressed miRNAs, including upregulated miR-26a-5p and miR-101-3p as well as downregulated miR-23b-5p, miR-339-3p, and miR-425-5p in AML-derived samples (Barrera-Ramirez et al., 2017); these candidate miRNAs might serve as biomarkers of AML and provide new insight into the pathogenesis and treatment of AML.

In addition, exosomes can be used as markers of diseases progression. Researchers reported exosome profiling in human ES (exudative seroma) for the first time, which was obtained from the lymphatic drainage implanted after lymphadenectomy in melanoma patients. They found that ES-derived exosomes have richer proteins compared with serum derived exosomes, including HSP90B, Annexin A1, and S100 A4, which are related to antigen presentation, the ER–phagosome pathway, and G2/M transition. In comparison with N1a patients, the number of ES-derived exosomes as well as the proteins associated with melanoma tumor cells significantly increased. Finally, they detected BRAFV600E mutation in ES-derived exosomal nucleic acids, which could serve as a factor to evaluate the risk of relapse in patients (García-Silva et al., 2019). In addition, Broggi et al. (2019) also analyzed the lymphatic exudate of metastatic melanoma patients undergoing lymphadenectomy (LAN) and demonstrated that exosomes carrying melanoma-related miRNAs or proteins could be applied as indicators of nodal metastatic spread.

## Application of MSC-Derived Exosomes in Bone Tissue Engineering

Many clinical diseases can lead to bone defects and reduced bone formation, including fractures (Matsumoto et al., 2010), bone tumors (Waning et al., 2019), osteoporosis (Cao et al., 2012), and so on. The commonly used treatments, such as bone transplantation, are still very limited. How to promote bone formation and repair bone defects has therefore always been a challenge faced by clinicians. Based on the cytobiology and materialogy, bone tissue engineering—now a significant research problem—is focused on promoting bone tissue regeneration by using seed cells, biological scaffolds, and bioactive factors (Bose et al., 2012). Previous studies have reported the important role MSCs play in repairing damaged organs and tissues through multiple mechanisms (Caplan and Dennis, 2006; Cao et al., 2012). BMSCs are most commonly used cells in stem cell researches due to their abundant sources and convenient separation methods (Morikawa et al., 2009). Recently scientists found that MSC-derived exosomes—the microvesicles mediating intercellular communication—have great potential in bone tissue engineering.

Zhang et al. demonstrated that MSC-Exosome/β-TCP complex scaffolds have a better repair effect on rat skull defects in comparison with pure β-TCP scaffolds. They found that the MSC-Exosomes could be internalized into HMBSs and promote their proliferation, migration, and differentiation *in vitro*. They then examined the gene expression in hBMSCs induced by exosomes, and the results suggested that the underlying mechanism by which exosomes stimulate osteogenic differentiation partly rely on the activation of the PI3K/Akt pathway (Zhang et al., 2016). Qi et al. (2016) indicated that hiPSC-MSC-Exos could upregulate the expression of RUNX2, COL1, and ALP and activate the differentiation of osteoblasts *in vitro*. They also conducted an *in vivo* experiment in mice with calvarial defects, and the results showed that hiPSC-MSC-Exos could stimulate bone formation in osteoporotic conditions. Moreover, the three-dimensional micro-CT images showed higher neovascularization in β-TCP + Exos group compared with β-TCP group. Taken together, these works suggested the potential application of MSC-Exos + β-TCP scaffolds in bone defects due to their effect of facilitating angiogenesis and osteogenesis (Qi et al., 2016). In another study, researchers produced two same-sized calvarial bone defects in SD rats and treated them with hydrogel and hydrogel + exosomes; the results showed that exosomes from BMSCs could significantly enhance the bone formation in rats in the aspects of micro-CT, histological examinations, HE, and Masson staining (Qin et al., 2016).

In addition to bone remodeling, angiogenesis is also extremely important for the maintenance of bone homeostasis and bone regeneration (Saran et al., 2014; Xie et al., 2014). Angiogenesis refers to the formation of blood vessels from microvascular endothelial cells through budding, bridging, and microvascular fusion, all of which aid the transportation of necessary oxygen, nutrients, and inorganic salts to bone via extensive networks (Adams and Alitalo, 2007). Previous studies have reported that MSCs-derived exosomes play a role in promoting angiogenesis in heart (Bian et al., 2014), renal injury, cutaneous repair (Zhang et al., 2015), skeletal muscle regeneration (Nakamura et al., 2015), limb ischemia repair, and so on. Likewise, in the process of fracture healing, new blood vessels can provide nutritional support, accelerate local metabolic rate, as well as improve the efficiency of bone reconstruction and the speed of fracture healing (Deschaseaux et al., 2009; Seebach et al., 2012). For example, MSC-derived exosomes could rescue delayed fracture healing in CD9^–/–^ mice and facilitate fracture healing in wild type mice; the mechanism is partly depended on the effect of MCP-1, -3, SDF-1, angiogenic factors and miRNAs (Furuta et al., 2016). In ischemic femoral head necrosis, it is also significant to promote angiogenesis and restrain ischemic necrosis areas (Glueck et al., 2007). Li H. et al. (2017) indicated that BMSC-ExosMU (exosomes secreted from HIF-1α-overexpressing BMSCs) could promote osteogenic differentiation of BMSCs through enhancing the expression of OCN and ALP *in vitro*. The MTT assay and cell-scratched wound evaluation also found that BMSC-ExosMU could promote proliferation, migration, and tube formation of HUVECs. Next, they conducted an *in vivo* experiment, and the results revealed that the SANFH (steroid-induced avascular necrosis of femoral head) model treated with BMSC-ExosMU was detected higher vessel density and denser trabecular tissue generation compared with the control group, suggesting the significant role of BMSC-ExosMU in promoting osteogenesis and angiogenesis (Li H. et al., 2017).

## Exosomes as Targeted Delivery Vehicles for Therapy in Skeletal and Extraosseous Disorders

In recent years, the application of pharmaceutical carriers in clinical diseases has received extensive attention, and this has particularly applied to nanoscale pharmaceutical carriers, such as liposomes, microparticles, nanoparticles, etc. They have several characteristics that contribute to their high delivery efficiency, including the capacity to encapsulate hydrophobic drugs, improve drug bioavailability, transport molecules with high specificity, and so on (Tan et al., 2010). Despite the advantages mentioned above, the artificial nano drug-loading systems still face many problems. For example, the carrier molecules are easily removed by antibodies as well as complement and coagulation factors. Besides, a toxic reaction cannot be avoided due to their synthetic lipid membranes. As natural endogenous nano-microvesicles, exosomes play important roles in intercellular signal transmission and material exchange. In contrast, exosomes can overcome multiple limitations of artificial nano delivery systems and are more suitable candidates for delivery vehicles. Firstly, the exosomes have higher security because they can evade the immune detection system; in this case the immune rejection is eliminated. They also have better tolerance, which helps to protect cargo from destruction, thus leading to a longer circulating half-life. Secondly, exosomes have a natural targeting capacity depending on their cell sources. Thirdly, the membrane of exosomes can be artificially modified to enhance their functions. Finally, exosomes can penetrate cytomembranes and biological barriers easily due to their nanometer size and specific surface molecules (Ei Andaloussi et al., 2013; Jiang and Gao, 2017).

### Exosome as Compounds Delivery System

At present, some chemotherapeutic drugs, such as doxorubicin and paclitaxel, can be effectively loaded into exosomes. Tian et al. (2014) indicated that exosome-encapsulated Dox (doxorubicin) can be integrated into breast cancer cells with high efficiency and significantly enhanced the therapeutic effect of Dox. They also evaluated the cardiac toxicity in tumor-bearing mice, a dose-dependent side effect of Dox (Minotti et al., 2004), and found that intravenously injected iExos-Dox has weaker cardiotoxicity compared with free Dox, suggesting that exsomes are ideal drug delivery vehicles with high safety and efficiency for targeted tumor therapy. Another study revealed that the PTX (Paclitaxel) incorporated into exosomes by mild sonication can maintain stability at various conditions for over a month and has significantly enhanced drug cytotoxicity compared with PTX alone (Kim et al., 2016). In addition to transporting chemotherapeutic drugs to target cells with high efficiency, exosomes can also greatly increase the effects of other drugs, such as anti-inflammatory agents. For example, Sun et al. (2010) found that the binding of curcumin to naturally existing nanoparticle exosomes can significantly enhance the curcumin’s anti-inflammatory activity through increasing its delivery into targeted macrophages, which leads to an improved anti-shock function in mice treated with LPS.

### Exosome as siRNA Delivery System

Currently, siRNA technology has been widely used in gene function research, clinical therapy, and other fields for the reason that it can induce targeted mRNA degradation and lead to post-transcriptional gene silencing (Burnett and Rossi, 2012; Crooke et al., 2018). However, siRNA-based gene treatment still faces many challenges, such as the poor cellular uptake and low stability in the circulating system. There is much evidence to suggest that the nanocarrier exosomes facilitate siRNA uptake into the targeted cells and improve its pharmacokinetics (Ozcan et al., 2015; Wang et al., 2017). It was reported that the exosome/TRPP2 siRNA complex can efficiently deliver TRPP2 (transient receptor potential polycystic 2) siRNA into FaDu cells and suppress its expression, resulting in the inhibition of the EMT (epithelial-mesenchymal transition) process and reduced invasion of FaDu cells (Wang et al., 2019a). Shokrollahi et al. (2019) treated human neuroblastoma cells (SH-SY5Y) with exosomes transfected by Hsp27 siRNA and evaluated the maturation of the human neuroblastoma cells. The data showed that the uptake of exosomes carrying siRNA HSP-27 by SH-SY5Y cells significantly inhibited their survival, clonogenic activity, and differentiation toward neuronal lineage (Shokrollahi et al., 2019). Shtam et al. (2013) used siRNA-loading exosomes to deliver RAD51-siRNA into HeLa cells and induce gene silencing, thereby inhibiting cell survival and proliferation. Exosomes can also be engineered into more suitable nanocarriers for carrying siRNA through modification. To enhance the targeting ability, Alvarez-Erviti et al. (2011) remolded the exosomes through attaching a neuron-specific RVG peptide to Lamp2b expressed on the exosomal membrane. The result revealed that the RVG exosomes were able to transport GAPDH siRNA into targeted neurons, microglia, and oligodendrocytes, and this resulted in downregulation of BACE1 gene expression, which plays a key role in Alzheimer’s disease (Alvarez-Erviti et al., 2011).

### Exosome as miRNA Delivery System

miRNAs are a group of non-coding single-stranded RNAs of approximately 22 nucleotides that exist in plant and animal cells (Bartel, 2004). A series of studies have shown that miRNAs can participate in cellular gene network regulation by silencing target mRNA, and they therefore play important roles in cell proliferation, differentiation, and apoptosis (Beavers et al., 2015). It has also been summarized that miRNAs play key roles in bone formation and may serve as the new breakthrough in the treatment of skeletal diseases (Hu et al., 2010). As previously mentioned, an exosome can transfer its cargo, such as miRNAs, into targeted cells and regulate their growth and differentiation (Thomou et al., 2017). For instance, brown fat-derived exosomes carrying specific miRNA could regulate liver through targeting Fgf21 (Chen and Pfeifer, 2017). It has been proposed that the exosome can be used as a vehicle for transporting functional miRNAs and exerting therapeutic action. The miRNAs have been proven to be involved in the pathogenesis of several neurodegenerative diseases, such as Alzheimer’s disease, Parkinson’s disease, etc. The delivery of miRNAs packed by exosomes to central nervous system is therefore widely used for the improvement of neurological functions (Ridolfi and Abdel-Haq, 2017). Katakowski et al. (2013) obtained exosomes carrying miR-146b through plasmid transfection, and they found that miR-146b can be delivered into tumor cells via exosomes, suppressing expression of EGFR and NF-jB proteins and leading to inhibition of glioma growth *in vitro*. They also demonstrated that M146-exo can significantly reduce tumor growth in the rat brain (Katakowski et al., 2013). Chen et al. (2018) demonstrated that the miR-223 transferred by BMSCs-exo can greatly protect hepatocytes from injury in autoimmune hepatitis. One study conducted by Yu et al. (2015) revealed that anti-apoptotic miRNAs like miR-19a can be delivered into cardiomyocytes via MSC^GATA–4^ (MSC overexpressing GATA-4)-derived exosomes and promote cardiac protection, which effect is connected with the inhibition of miR-19a target PTEN as well as activation of the Akt and ERK signaling pathway.

## Conclusion and Perspective

Since the first discovery of exosomes released by mature erythrocytes in 1983, this topic has attracted much attention from researchers because of their inherent properties. Exosomes are a class of extracellular vesicles sized 30–100 nm that contain cholesterol, sphingomyelin, ceramide, and other lipids on the surface that can be secreted by a variety of cells, including BMSCs, osteoclasts, osteoblasts, osteocytes, dendritic cells, epithelial cells, tumor cells, etc. In the recent years, researchers have developed various techniques to isolate exosomes based on their physical, chemical, and biological properties, and different methods have their unique advantages and disadvantages which may influence the product quality and following analysis.

With the development of molecular biotechnology, many researchers have tended to investigate the bone metabolism and skeletal disorders from a paracrine perspective, among which the exosome-mediated intercellular communication is rapidly gaining increasing attention. Bone-derived exosomes can regulate cell apoptosis, proliferation, and differentiation through multiple pathways, thus exerting important effects during physiological and pathological processes, such as bone remodeling, bone loss, fracture healing, and so on. This review has summarized the role of exosomes derived from BMSCs, osteoclasts, osteoblasts, and osteocytes in skeletal metabolism. The multiple effects of bone-derived exosomal miRNAs in bone remodeling have also been listed in Table 1. Besides, the bone-derived exosomes are found to participate in development of extraosseous diseases. For example, bone-derived exosomes could promote or suppress tumor growth through regulating genes expression, signaling pathways, angiogenesis, and so on. There are also numerous experiments illustrating that bone-derived exosomes are able to protect functional cells from apoptosis, thereby facilitating nerve repair, preventing nephropathy, preserving myocardial functions, and so on. It is reasonable to believe that bone-derived exosomes are highly relevant to the exploration of mechanisms of extraosseous diseases based on their ability to reach distant tissues and extraosseous systems.

**TABLE 1:** The role of bone-derived exosomal miRNAs in bone remolding.

Exosomal miRNA	Cells	miRNA expression	miRNA function
miR-27a	BMSCs	High	Promote osteogenic differentiation of osteoblasts (Qin et al., 2016)
miR-206a			
miR-196a			
miR-214	Osteoclasts	High	Inhibit osteoblasts function Promote RANKL induced osteoclasts differentiation (Zhao et al., 2015; Sun et al., 2016)
miR-30d-5p	Mineralizing MC3T3 cells	High	Inhibit osteoblasts differentiation (Xie et al., 2017)
miR-133b-3p			
miR-140-3p			
let-7	Mineralizing MC3T3 cells	High	Promote osteoblasts differentiation (Xie et al., 2017)
miR-335-3p			
miR-378b			
miR-218	Osteocytes	Low	Inhibit osteoblastic differentiation (Zhang et al., 2014; Qin et al., 2017)
miR-1192	Mineralizing osteoblasts (MOBs)	High	Promote bone marrow stromal cell (ST2) differentiation to osteoblasts (Cui et al., 2016)
miR-680			
miR-302a			

In recent years, scientists have discovered the important role of exosomes in transmitting information between cells. The contents of the exosomes can reflect the physiological and pathological processes in cells, which can be released into various body fluids and microenvironments, thereby transmitting carried signals to distant tissues and cells (Yoshida et al., 2019). Exosomes can therefore be used for diseases diagnosis. In addition, the microvesicular structure of the exosomes can protect their contents from degradation, which demonstrates their stability in body fluids and enormous potential in the diagnosis and surveillance of diseases (Kourembanas, 2015). Bone-derived exosomes have become a research hotspot in the context of their use as biomarkers for clinical diseases, such as osteoporosis, MDS, AML, and so on; this provides a great opportunity for the development of a novel and sensitive diagnosis method.

BMSCs are one of the most commonly used seed cells in tissue engineering. A series of studies have shown that BMSCs have the ability to repair bone defects (Rackwitz et al., 2012), but there still exist some problems in terms of the immune response, ethics, and so on. Recently, it has been reported that BMSCs release exosomes containing signaling molecules through paracrine mechanisms to promote tissue repair (Liang et al., 2014). Therefore, scientists have started to focus on the application of BMSC-derived exosomes in bone tissue engineering. It has been found that BMSC-derived exosomes have the function of regulating osteoblasts and osteoclasts as well as promoting bone formation and neovascularization, and exosomes thus have great application prospects and research value in the field of bone tissue engineering. In addition, the potential use of exosomes as drug delivery systems for disease treatment has gained significant interest from the scientific community since they are of a nano-size and have strong penetrability and hypotoxicity as well as low immunogenicity and cell targeting properties. However, the clinical application of bone-derived exosomes still faces several challenges. The exosome extraction method currently used is not efficient enough to be applied in clinic; it is therefore imperative to design a strategy to increase the yield and purity of exosomes. Besides, the content and function of exosomes from different sources differ greatly, and the genetic information contained in exosomes is thus still not fully elucidated. There is consequently still a long way to go before exosomes can be widely used in clinic.

In summary, this article has provided an overview of the effect and mechanism of bone-derived exosomes in skeletal metabolism and extraosseous diseases, and it has opened up new perspectives for exosomes serving as biomarkers and drug delivery carriers. We have reason to believe that, with the further development of biotechnology and in-depth exploration of exosomes, the role of bone-derived exosomes in clinical diagnosis, monitoring, and treatment will be fully utilized.

## Author Contributions

HL wrote the manuscript and designed the figures. YX, QG, and YH revised the manuscript. XL provided critical feedback and helped to shape the manuscript. All authors listed have made a substantial contribution to the work.

## Conflict of Interest

The authors declare that the research was conducted in the absence of any commercial or financial relationships that could be construed as a potential conflict of interest.
